# Classic Testicular Seminoma in Men Aged 50 Years or Over: A Case Report and Review of the Literature

**DOI:** 10.1155/2019/4391015

**Published:** 2019-11-24

**Authors:** Rawad Abou Zahr, Khalil Chalhoub, Imad Matta, Sawsan Horani, Zarouhie Bedoyan, Georges El Hachem, Imad Ghantous, Michel Jabbour

**Affiliations:** ^1^Department of Urology, University of Balamand, Saint George Hospital University Medical Center, Beirut 1100 2807, Lebanon; ^2^University of Balamand, Faculty of Medicine and Medical Sciences, Beirut 1100 2807, Lebanon; ^3^Department of Pathology, University of Balamand, Saint George Hospital University Medical Center, Beirut 1100 2807, Lebanon; ^4^Department of Hematology-Oncology, University of Balamand, Saint George Hospital University Medical Center, Beirut 1100 2807, Lebanon

## Abstract

Testicular cancer is the most frequent solid tumor detected in young adult men. Germ cell tumors (GCTs), particularly seminomas, are the most common type of testicular neoplasms seen in that age population. Most publications have reported decreasing incidence of GCTs in patients above forty years of age. Since the biologic activity of seminomas appears similar across ages, recommended management of senior adults involves a multimodal therapy of radical inguinal orchiectomy with radiation or cytotoxic treatment as needed. Attenuating chemotherapy dosages are critical to ensure better tolerability of associated adverse events. Here we report a case series of 2 men older than fifty years of age with metastatic testicular seminoma. We aim to emphasize a rare clinical entity encountered in the senior adult population.

## 1. Introduction

Testicular GCTs account for most testicular cancers in young adult men and have a peak incidence in the second and third decades of life [1, 2]. Of all reported testicular cancers in the United States, more than 95% are predominantly GCTs, half of which are seminomas [2, 3]. Globally, seminoma incidence rates were highest in European and Northern American countries and lowest in Asian and African countries [4]. The highest incidence rates were witnessed in Norway and Denmark (5.5/100,000 man-years), and the lowest rates were encountered in India and Uganda (<0.5/100,000 man-years) [4]. The World Health Organization's (WHO) 2016 updated classification of testicular GCTs now categorizes them based on histopathology into seminomas, non-seminomas, and spermatocytic tumors [5].

Despite their high incidence in young adults, testicular GCTs are rarely encountered in senior adults. Only less than 4% of patients with testicular GCTs are above sixty five years of age [6]. Recent epidemiologic studies highlight an increased incidence of seminomas across all age groups as well as an older age at diagnosis [7]. Mean age of diagnosis has shifted from 34 to 39 years for seminomas, as opposed to 26 to 31 years for non-seminomas [7]. In most clinicopathological studies seminomas, non-seminomas, and spermatocytic tumors account for 50–55%, 45–50% and 1% of testicular cancers, respectively [7, 8]. The biological phenotype and malignant potential remain the same across age groups suggesting that similar treatment regimens should be pursued [9].

Macroscopically, seminomas are well circumscribed, tan to pale yellow lesions with necrotic or hemorrhagic foci. The cells are arranged into nests and sheets with intercepting thin fibrovascular septa which have lymphocytes and sometimes syncytiotrophoblasts.

Cells have clear or eosinophilic cytoplasm with large nuclei and prominent nucleoli and they are immunoreactive to SALL4, OCT3/4, C-KIT, D2-40, and SOX17 [9]. Patients with seminomas can have elevated *β*-hCG and LDH but never AFP [8, 10]. Only about 30% of patients can have elevated *β*-hCG. LDH is a positive non-specific marker that reflect tumor burden [10].

Common metastatic sites of testicular seminomas involve the lungs and the liver. Rarely, it can also metastasize to the bones and the brain [11]. Even at advanced stages, testicular seminomas can still have high cure rates. The most common stage of presentation, stage I seminoma, has a cure rate above 95% [12]. Considering all stages, the survival rate is 86% and 71% for the 5- and 10-year survival rate, respectively [9]. Important predictors of metastasis involve lymphovascular invasion by the primary tumor while predictors of relapse involve tumor size (>4 cm) and invasion of the rete testis. When both relapse predictors are absent, there is only a 6% risk of recurrence [12, 13].

## 2. Case 1

A 56-year-old male was presented to our facility for testicular pain of 3 weeks duration associated with progressive increase in the size and swelling of the right testicle. Physical examination revealed enlarged and rigid right testicle associated with tenderness.

**Figure 1:**
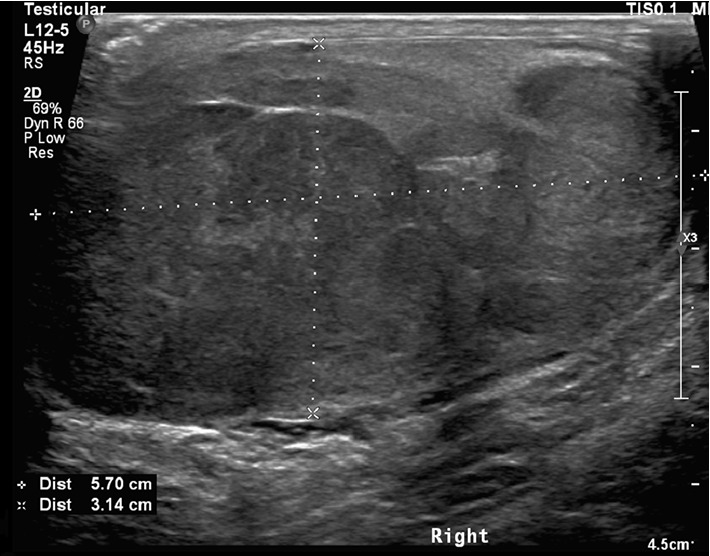
Ultrasound of right testicle.

Ultrasonography of the testicles revealed a hypoechoic heterogeneous well circumscribed lesion measuring more than 5.7 × 3.1 × 4.1 cm with nodularity and vascularity (Figure 1).

Metastatic work up included the Chest Abdomen Pelvis CT scan which revealed multiple enlarged retroperitoneal, para-aortic, and aorto caval lymphadenopathies. The largest lymph node was found in the left para aortic space with size 3 × 5 cm. Tumor markers were done upon admission showing negative AFP (2.31 ng/ml), slightly elevated BHCG (9.91 mIU/ml) and increased LDH levels (801 U/L). The patient underwent a right radical inguinal orchiectomy. The gross histology study demonstrated parenchyma of the testes occupied by a tan yellow, more or less well demarcated soft tumor mass measuring 5 × 2 × 2 cm with areas of yellow necrosis. Diagnosis concluded classic seminoma stage pT2, with lymphovascular invasion and negative spermatic cord margins. No evidence of invasion of the rete testis, tunica albuginea, tunica vaginalis, or epididymis. Immunostaining revealed positive expression of PLAP and SALL4 antibodies by tumor cells and negative anti-cytokeratin antibody.

Patient was diagnosed with stage IIB classical seminoma, he was started on cytotoxic chemotherapy consisting of 1 cycle of BEP protocol (Bleomycin, Etoposide, and Cisplatin) followed by 3 cycles of EP (Etoposide and Cisplatin) without Bleomycin because of pulmonary toxicity. A spirometry was performed since the patient was at high risk for pulmonary complications secondary to his smoking status and showed a 25% decrease in DLCO (Diffusing capacity of the Lungs for Carbon Monoxide), prompting bleomycin interruption [14]. He received 5 days of granulocytes colony stimulating factors (G-CSF) to prevent febrile neutropenia. The LDH normalized at the end of the first cycle. The Chest Abdomen Pelvis CT scan was repeated after the fourth cycle revealing an excellent partial response with persistence of 2 lymph nodes of 1.8 and 1.9 cm, with necrotic centers. The 4 cycles of chemotherapy were smooth without any renal, oto- or neuro-toxicity. He did not have any episode of febrile neutropenia. The DLCO re-increased by 15%, without reaching the pre-chemotherapy value. Since the remaining lymph nodes were less than 3 cm, close follow up with CT scan was scheduled on a bi-yearly basis for the first year and then yearly once for five years. To date the patient is being followed up 24 months after diagnosis and is doing well with no evidence of recurrence.

## 3. Case 2

A 65-year-old male patient, previously healthy, presented to our emergency department for a new onset of testicular pain following by swelling of his right testicle that started 4 weeks ago.

On physical examination, the patient had an apparent large and rigid right testicle with moderate tenderness upon palpation.

**Figure 3:**
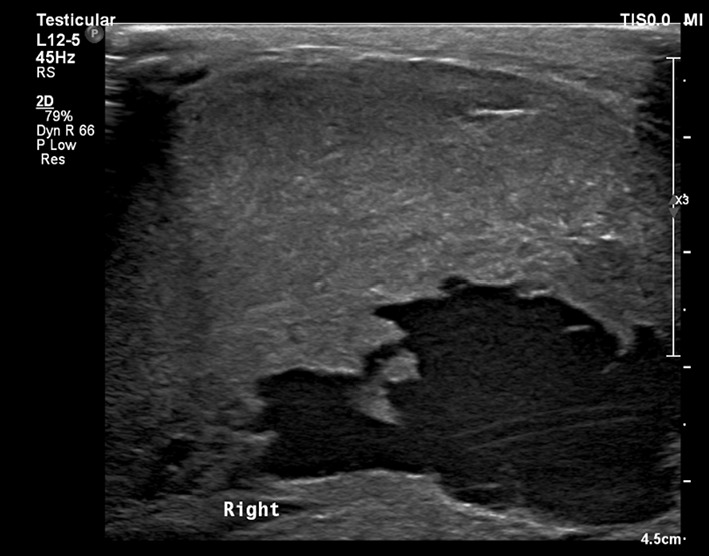
Ultrasound of right testicle.

Ultrasonography of the testicles revealed a very large heterogenous vascularized mass in the right scrotum showing cystic components and containing fine echoes (Figure 3). The patient was admitted for further diagnosis and management. On the ward, tumor markers were done showing normal AFP and BHCG levels, 1.65 ng/ml and 3.63 mIU/ml respectively, and an elevated LDH level of 891 U/L.

The Chest Abdomen Pelvis CT scan was done as metastatic workup and showed few well circumscribed subpleural solid lung nodules measuring 1.4 cm in the apical segment of the right upper lobe, 2.5 × 2.8 cm in the anterior basal segment of right lower lobe and 3.3 × 1.6 cm in the posterior segment of the left lower lobe. Bulky necrotic retroperitoneal adenopathy was also noted in the portacaval region measuring up to 3.5 × 3 × 3 cm consistent with nodal metastases. After confirming the diagnosis, the patient underwent right radical orchiectomy. The final pathology came out as Classic Pure Seminoma, measuring 13 cm in largest dimension with extensive areas of necrosis and cystic degeneration. The Tumor focally invaded the tunica albuginea and reached the surface of the inked visceral layer of the tunica vaginalis. Invasion of the spermatic cord was also noted. Resection margin of the spermatic cord was free on neoplasia. Final diagnosis established a Classic Pure Seminoma stage pT3 N2 M1a.

The patient was classified to have good prognosis according to the International Germ Cell Cancer Collaborative Group (IGCCCG) risk classification and was started on 3 cycles of BEP protocol. His chemotherapy sessions were uneventful and smooth with no reported complications. A similar protocol of follow up with the Chest Abdomen Pelvis CT scan was scheduled on a bi-yearly basis for the first year and then once yearly for five years. To date, the patient is doing well 14 months post diagnosis and is currently being followed up as per the assigned protocol.

## 4. Discussion

Testicular GCTs is the number one cancer detected in young male adults, accounting for 21% of diagnosed invasive cancers in patients between 20 and 40 years of age. GCTs are rarely encountered in prepubertal males as well as senior adult men [15]. Most articles reviewed in the literature report showed declining incidence of testicular GCTs beyond the age of 40 [15, 16].

Histologically, studies showed that seminomas predominate in patients above fifty years old, while non-seminomas predominate in younger counterparts [16].

The clinical characteristics of seminomas in the elderly has had conflicting findings in the literature. The most common presenting symptom in older adults as well as young adults is a palpable, and painless scrotal mass that can be associated with discomfort [8]. Similarly, our patients were presented to our facility for a right testicular mass. According to Berney et al., seminomas in patients aged 50 and older tend to present at advanced stages and demonstrate aggressive behavior. Fifty cases were reviewed, and the results show that in senior adults with GCTs have a significantly larger macroscopic size and more frequent invasion of the rete testis and vascular invasion, as well as less frequent intratubular germ cell neoplasia [16].

One of the earliest publications on seminomas defines them as malignant GCTs that arise from the germinal epithelium of testicles and originate from precursor cells of the spermatogonium [17]. Rarely, however; about 5% of primary seminomas can also arise in extragonadal sites such as the pineal gland of the brain, the anterior mediastinum, and the retroperitoneum [18]. Primary GCTs in senior adults most commonly arise in the mediastinum followed by the retroperitoneum, and the CNS, while primary GCTs in young adult men are most commonly detected in the cranium [18].

A recent large-scale study by the Cancer Registration Committee of the Japanese Urological Association (2016) states that only 11% (123 out of 1119) of patients above 50 years old developed primary GCTs and 74.8% (91) of the GCTs in that age category were seminomas. 79.5% of the seminomas were stage I, 6.6% were stage II, and 13.9% were stage III according to the Union for International Cancer Control (UICC) classification. Furthermore, 84% of reported metastatic GCTs had a good prognosis as per the International Germ Cell Cancer Collaborative Group (IGCCC) vs. 16% and 0% had intermediate and poor prognosis, respectively. Survival, in months, included 72, 20 and 0 patients at 24, 48, and 60 months, respectively [19].

Wheater et al. studied 60 patients aged 60 and above. 44 patients were reported to have pure seminoma (73%) while the remaining 16 had non seminomatous and mixed tumors. Among the 44 patients with seminomas, 33 patients had stage I seminoma, 2 had stage II seminoma, and the rest had stage III seminoma. The median survival was 194 months for those with stage I seminoma and 70 months with metastatic disease [20].

In a case report written by Foell et al., an 86-year-old, the oldest patient in literature, with classic seminoma was compared with 4 patients under the age of 50. The authors state that the histologic features, biologic activity, and phenotype of the seminoma were similar and suggested following similar treatment algorithms [9]. The primary therapy for testicular seminoma is radical inguinal orchiectomy as it allows for accurate staging and histologic diagnosis of the tumor. Scrotal violations are avoided at all cost as they increased the risk of seeding of the scrotum and damaging regional lymphatic drainage [2].

Adjuvant therapy depends on staging but usually involves a multimodal approach. For stage I seminomas, active surveillance or carboplatin are recommended. Stage IIA and IIB seminomas are managed with dog-leg radiotherapy; the irradiation of the para-aortic lymph nodes with the ipsilateral iliac lymph nodes. Chemotherapy including 4 cycles of EP protocol (etoposide and cisplatin) or 3 cycles of BEP protocol (bleomycin, etoposide, and cisplatin), are used for stage IIA, IIB, IIC as an alternative to radiation as well as stage III seminomas [21]. For stage II seminomas, both radiotherapy and cisplatin-based chemotherapy are reported to be equally effective with the latter favored due to lower relapse rates. To note, carboplatin monotherapy has also been tried in stage II seminomas with reports of worse outcomes, however; when combined with radiotherapy it has been proven promising [21].

Testicular GCTs are highly responsive to chemotherapy and radiotherapy, thus; making a good outcome highly likely [16]. Both modalities, however, carry risks. Radiation runs the risk of secondary malignancies and cardiotoxicity, while the BEP protocol can cause cardiopulmonary toxicity, neurotoxicity, nephrotoxicity, and infertility as well as secondary cancer [3, 22]. Our patient was managed by a right radical inguinal orchiectomy and completed 1 cycle of the BEP protocol but was later switched to 3 cycles of EP as he developed a pulmonary reaction to Bleomycin at the start of his second cycle.

Most recent studies recommend following identical treatment algorithms, owing to the similar phenotype of seminomas in both age populations, but it is important to discuss factors hypothesized to lower treatment outcomes in senior adults. The age-related functional decline or associated comorbidities seen in this age bracket renders them less tolerable to the adverse effects and toxicities associated with chemotherapy. Prophylactic attenuation of chemotherapy dosage in senior adults is essential [23].

Those with contraindications to Bleomycin can alternatively receive 4 cycles of EP if they are in the good prognosis group and 4 cycles of VIP if they are in the intermediate or poor prognosis groups. The alternative EP and VIP regimens have been reported to have equivalent success rates, though the former regimen has been associated with lower rates of disease free survival [20, 22]. Wheater et al. modified the conventional BEP regimen by delivering a single empirically-reduced dose of 30,000 International Units of Bleomycin per cycle on the second day, without evidence of significant pulmonary toxicity [20]. To note, other trials have attempted to reduce Etoposide from the conventional dose of 500 mg/m^2^to 360 mg/m^2^ with complete exclusion of Bleomycin but reported poorer outcomes [20].

For instance, advanced age and associated declining renal function in senior patients pose a 5% risk of Bleomycin pulmonary toxicity due to advanced age alone and a 20% risk when pooled with impaired renal function [19]. Thus, cautious selection of chemotherapeutic agents as well as dosages is necessary. According to Beyer et al., the BEP protocol is the mainstay of chemotherapy for senior patients with metastatic disease in good, intermediate, and poor prognosis groups [22].

Another concern is chemotherapy-induced nephrotoxicity particularly associated with cisplatin. The primary measures of mitigating cisplatin-related nephrotoxicity involve the combination of adequate hydration (≥3 L/day) and osmotic diuresis with mannitol during chemotherapy. Alternatively, patients with declining renal function could substitute cisplatin with carboplatin. Carboplatin dose setting based on renal function after target AUC has been established is also recommended [24].

The optimal salvage therapy for elderly patients with seminomas who relapse after surveillance, adjuvant carboplatin or adjuvant radiotherapy remains unknown. Several combinations of chemotherapy were used as a first-line salvage treatment, including conventional dose chemotherapy (CDCT) and high dose chemotherapy (HDCT). HDCT was reserved for patients with unfavorable prognostic factors and high risk of failure of salvage therapy. CDCT regimens included 4 cycles of VIP, TIP or VeIP, and HDCT which comprised of 2–3 sequential cycles of etoposide and carboplatin. In the second and third salvage therapy setting, other agents such as oxaliplatin, gemcitabine, and paclitaxel have been recommended as single agent or combination chemotherapy [21, 22].

If residual seminoma masses present post chemotherapy or radiotherapy, close monitoring with tumor serum markers and imaging is recommended, especially if residual masses are >3 cm. The Fluorodeoxyglucose (FDG)-PET scanning is a reliable imaging modality with high prognostic value for residual masses post treatment. Patients with positive FDG-PET scans for residual masses should get biopsied. Based on histology, further treatment options could include observation, chemotherapy, radiation or surgical resection [25].

Follow up protocols vary based on staging and consist of active surveillance, tumor markers, and imaging (the chest abdominopelvic CT, the brain CT and CXR) [12]. Testicular ultrasound is reserved for ambiguous exams [26]. For stage I seminoma, in the first 2 years post-surgery, tumor markers test and physical exam should be conducted every 4 months, and abdominopelvic CT and CXR every 6 months. In years 3–5, the physical exam and tumor markers test are to be done once per year and abdominopelvic CT on the third and fifth year. For metastatic seminoma, in the first 2 years, tumor markers test, physical exam, and CXR are to be done every 3 months, abdominopelvic CT every 6 months, and the brain and the chest CT every 12 months. For years 3–5, tumor markers, physical exam, and CXR should be done every 6 years, and the chest abdominopelvic CT and the brain CT every 12 months [12].

## 5. Conclusion

Testicular GCTs are neoplasms encountered frequently in young adults and are rarely seen in the senior adult population. Histologically, seminomas predominate in patients over fifty years old and have retained the phenotype, biologic activity, and malignant potential. Seminomas remain curable and have good prognosis in senior adults even at advanced stages. Treatment regimens similar to younger patients can be employed as long as chemotherapy dosages are empirically reduced, and precautions are taken to mitigate chemotherapy-related toxicities.

## Figures and Tables

**Figure 2:**
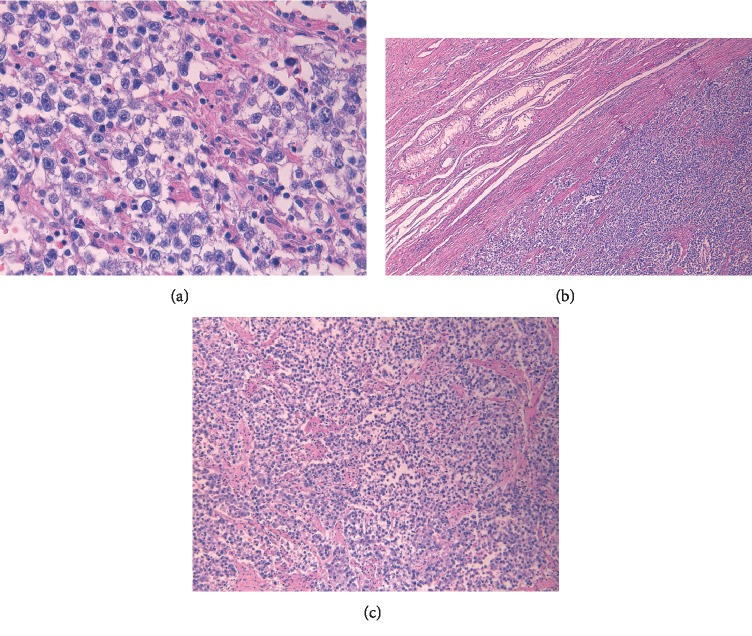
(a): High-power view shows round to polyhedral cells with distinct cell borders, and sparse lymphocytic infiltrate within fibrous septa, (b): Low-power view of the tumor (right) with background intratubular germ cell neoplasia (upper left), (c): Medium-power view of the tumor shows sheets of uniform clear cells divided into poorly demarcated lobules by delicate fibrous septa.
